# Genetic glycoengineering in mammalian cells

**DOI:** 10.1016/j.jbc.2021.100448

**Published:** 2021-02-20

**Authors:** Yoshiki Narimatsu, Christian Büll, Yen-Hsi Chen, Hans H. Wandall, Zhang Yang, Henrik Clausen

**Affiliations:** 1 Department of Cellular and Molecular Medicine, Faculty of Health Sciences, Copenhagen Center for Glycomics, University of Copenhagen, Copenhagen, Denmark; 2 GlycoDisplay ApS, Copenhagen, Denmark

**Keywords:** glycoengineering, glycome, glycosyltransferase, cell-based glycan array, glycoengineered organoids, glycosylation, glycoprotein therapeutics, glycan-binding protein, adhesin, lectin, ADCC, antibody-dependent cell cytotoxicity, CHO, Chinese hamster ovary, CRISPR, clustered regularly interspaced short palindromic repeat, EPO, erythropoietin, ES, embryonic stem, GBP, glycan-binding protein, GT, glycosyltransferase, HA, hemagglutinin, HDR, homology-directed repair, KI, knock-in, KO, knockout, MMEJ, microhomology-mediated end joining, NHEJ, nonhomologous end joining, PAM, proto-spacer adjacent motif, TALEN, transcription activator-like effector nuclease, TR, tandem repeat, ZFN, zinc-finger nuclease

## Abstract

Advances in nuclease-based gene-editing technologies have enabled precise, stable, and systematic genetic engineering of glycosylation capacities in mammalian cells, opening up a plethora of opportunities for studying the glycome and exploiting glycans in biomedicine. Glycoengineering using chemical, enzymatic, and genetic approaches has a long history, and precise gene editing provides a nearly unlimited playground for stable engineering of glycosylation in mammalian cells to explore and dissect the glycome and its many biological functions. Genetic engineering of glycosylation in cells also brings studies of the glycome to the single cell level and opens up wider use and integration of data in traditional omics workflows in cell biology. The last few years have seen new applications of glycoengineering in mammalian cells with perspectives for wider use in basic and applied glycosciences, and these have already led to discoveries of functions of glycans and improved designs of glycoprotein therapeutics. Here, we review the current state of the art of genetic glycoengineering in mammalian cells and highlight emerging opportunities.

Engineering glycosylation in cells and organisms has a long history (1, 2, 3), and yet rational genetic engineering of the glycosylation capacities in mammalian cells may only be off to a start (4) (Fig. 1). The glycosylation machinery in cells involves a large and complex metabolic network of enzymes and accessory proteins that orchestrate the synthesis of different types of glycans found on glycolipids, glycoproteins, proteoglycans, and as oligosaccharides. Over 200 distinct glycosyltransferase (GT) genes contribute to glycosylation in mammalian cells, and at least 173 of these GTs function in at least 16 distinct glycosylation pathways to assemble the great diversity of glycan structures found on glycoproteins, glycolipids, and proteoglycans (5). Rational engineering of glycosylation in mammalian cells can now be performed with a high degree of confidence in predicted outcomes, and genetic engineering is one approach to studying the biology of glycans that takes origin in a single cell. However, challenges still exist with isoenzymes for which unique functions are poorly characterized and partial overlaps in properties need to be considered.

**Figure 1:**
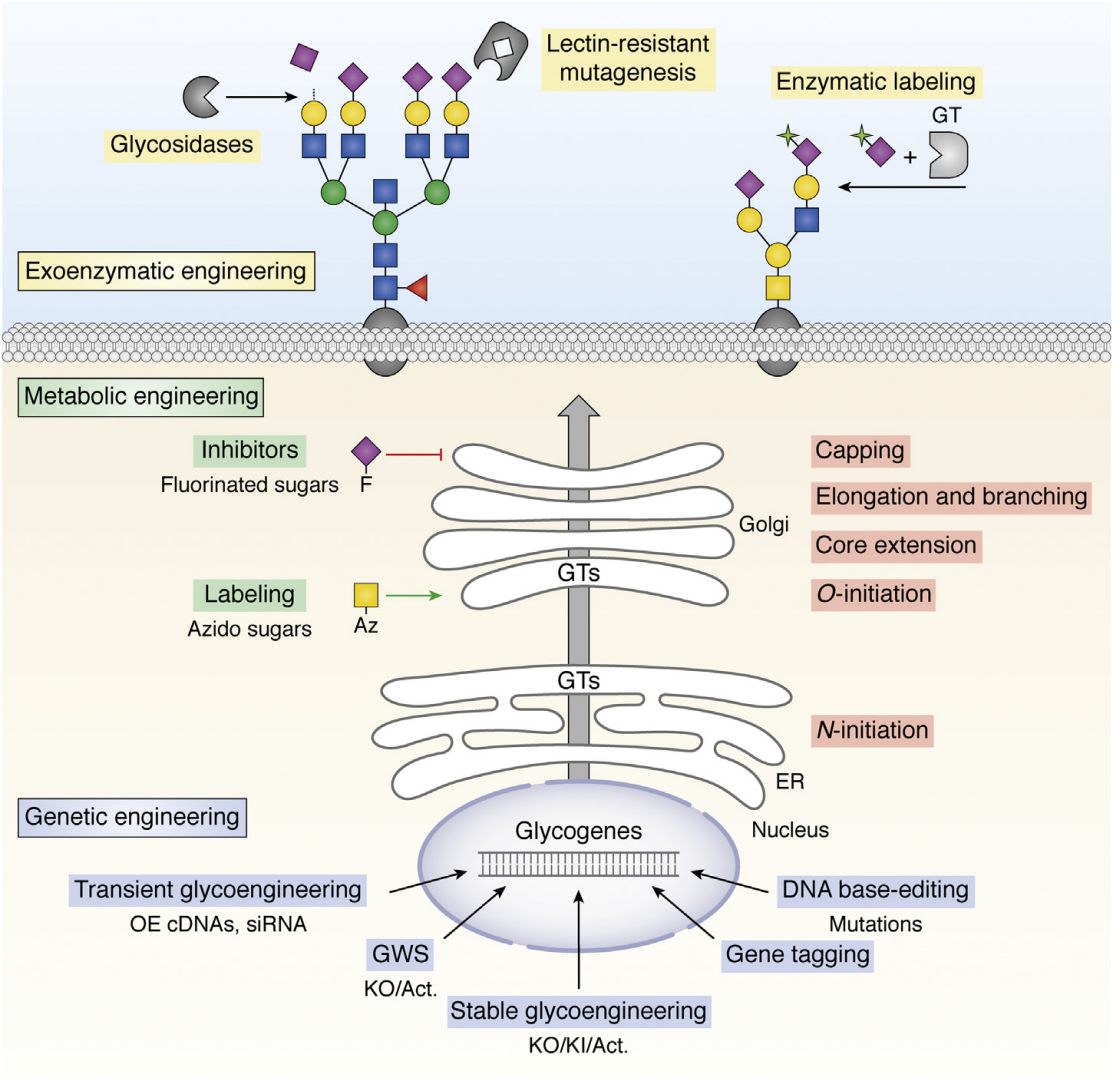
**Overview of glycoengineering strategies.** Basic principles for approaches available to modulate the cellular glycosylation processes and the glycome are illustrated. Extracellularly, glycans may be modulated by more or less selective endo-/exo-glycosidases (sialidases, galactosidases, PNGase, etc.) (117), and chemoenzymatic labeling methods utilizing, *e.g.*, glycosyltransferases (GTs) may be applied to install natural or unnatural substrates on cell surface glycans (6, 337). Use of cytotoxic lectins often in combination with mutagenesis may enable selection of mutant cells with loss/gain of distinct glycosylation features (10, 338). A growing number of unnatural sugar mimetics can be applied for metabolic engineering (6, 23), including glycosylation inhibitors (*i.e.*, fluorinated sugar analogues) (235, 339) or functionalized sugars (*i.e.*, azido, Az, sugars) that enable conjugation chemistries for use in glycan imaging or reprogramming of their interactions (20, 160). Genetic engineering of glycosylation may be performed by overexpression (OE) of GTs using cDNA plasmid transfection and/or siRNA for silencing of endogenous GTs (6). More extensive and stable glycoengineering takes advantage of precise gene engineering for combinatorial KO/KI/Act (activation) of GTs, and this strategy is the main focus of this review. Genome-wide KO/Act screens (GWS) may be used for discovery and dissection of GTs and other genes affecting glycosylation (Table 1), and endogenous GTs may be mutated, *e.g.*, to mimic disease mutations or enable use of unique substrates, or tagged, *e.g.*, by insertion of antibody tags or fluorescent proteins (169).

Genetic engineering of glycosylation in mammalian cells has contributed tremendously to the current view of the genetic and biosynthetic regulation of the cellular glycome and highlighted important functions of glycans in development, health, and disease (1, 2, 3, 5, 6). Early on mammalian mutant cell lines with loss of GT genes or other genes affecting glycosylation were generated by random mutagenesis and selection for lectin resistance (7, 8, 9, 10), and later targeted knockout (KO) of GT genes in animals by homologous gene recombination strategies in embryonic stem (ES) cells opened up rational design of glycoengineered models (1, 2, 3). These models informed about GT genes that are essential for early development of the embryo and genes with important functions for normal health and diseases, but also of many GT genes that at first pass at least appeared to be dispensable for normal development and health. These studies also highlighted that some steps in the glycosylation pathways in mammalian cells are regulated by a single GT with loss of function of such genes leading to global changes in the glycan structures produced. However, these studies also revealed glycosylation steps that are regulated by multiple isoenzymes with at least partly redundant functions, where loss of function of a single isoenzyme gene may lead to no or only subtle effects on the glycans (11, 12, 13, 14, 15).

A major obstacle for the wider use of genetic engineering of glycosylation in mammalian cells has until recently been the lack of simple and efficient means to design and conduct rational and combinatorial engineering of genes. In lieu of this, considerable efforts have been devoted to engineering glycosylation in other eukaryotic cells such as yeast, plants, and insects where tools for genetic engineering were more readily available (16, 17, 18, 19). Engineering of glycosylation has also been sought by a wide range of nongenetic strategies including chemical and enzymatic approaches (Fig. 1) (6, 20, 21, 22, 23). The primary objectives for genetic engineering of glycosylation were devoted to glycoprotein therapeutics, and the first targeted KO engineering in a mammalian cell involved a tour-de-force effort performed using two rounds of targeted homologous recombination in Chinese hamster ovary (CHO) cells to knockout the two copies of the α6-fucosyltransferase (*fut8*) gene. FUT8 directs core fucosylation of N-glycans and the CHO *fut8* KO cells enabled production of afucosylated IgG with greatly improved antibody-dependent cell cytotoxicity (ADCC) (24, 25). However, the huge efforts required with this strategy limit broader use for engineering mammalian cell lines.

A new era for glycoengineering emerged with the introduction of the facile nuclease-based gene-editing strategies at the turn of the millennium (4, 27). The zinc-finger nucleases (ZFNs), transcription activator-like effector nucleases (TALENs), and clustered regularly interspaced short palindromic repeats with the CRISPR-associated protein 9 (CRISPR/Cas9) tools now allow for rational designed gene engineering events in mammalian cells with high precision, speed, and low cost. Engineering events in cells can be stacked with multiple gene KOs in combination with knock-ins (KIs) to achieve almost any desirable design of the cellular glycosylation capacities. These new opportunities are beginning to thrive, and engineering of glycosylation in cell lines is no longer only focused on meeting interests in improved glycoprotein therapeutics. Already a remarkable diversity in the use of glycoengineering has emerged. Here, we focus on these new advances and discuss the use of strategies to comprehensively engineer and dissect glycosylation in mammalian cell lines, which we define as rational genetic engineering of glycosylation. A number of excellent reviews have already summarized and discussed progress with engineering glycosylation in different species including yeast, insects, and plants and the special opportunities these provide for production of glycoprotein therapeutics (6, 16, 17, 18, 19, 20, 21, 22, 28, 29, 30).

## Glycosylation processes in cells

**Figure 2:**
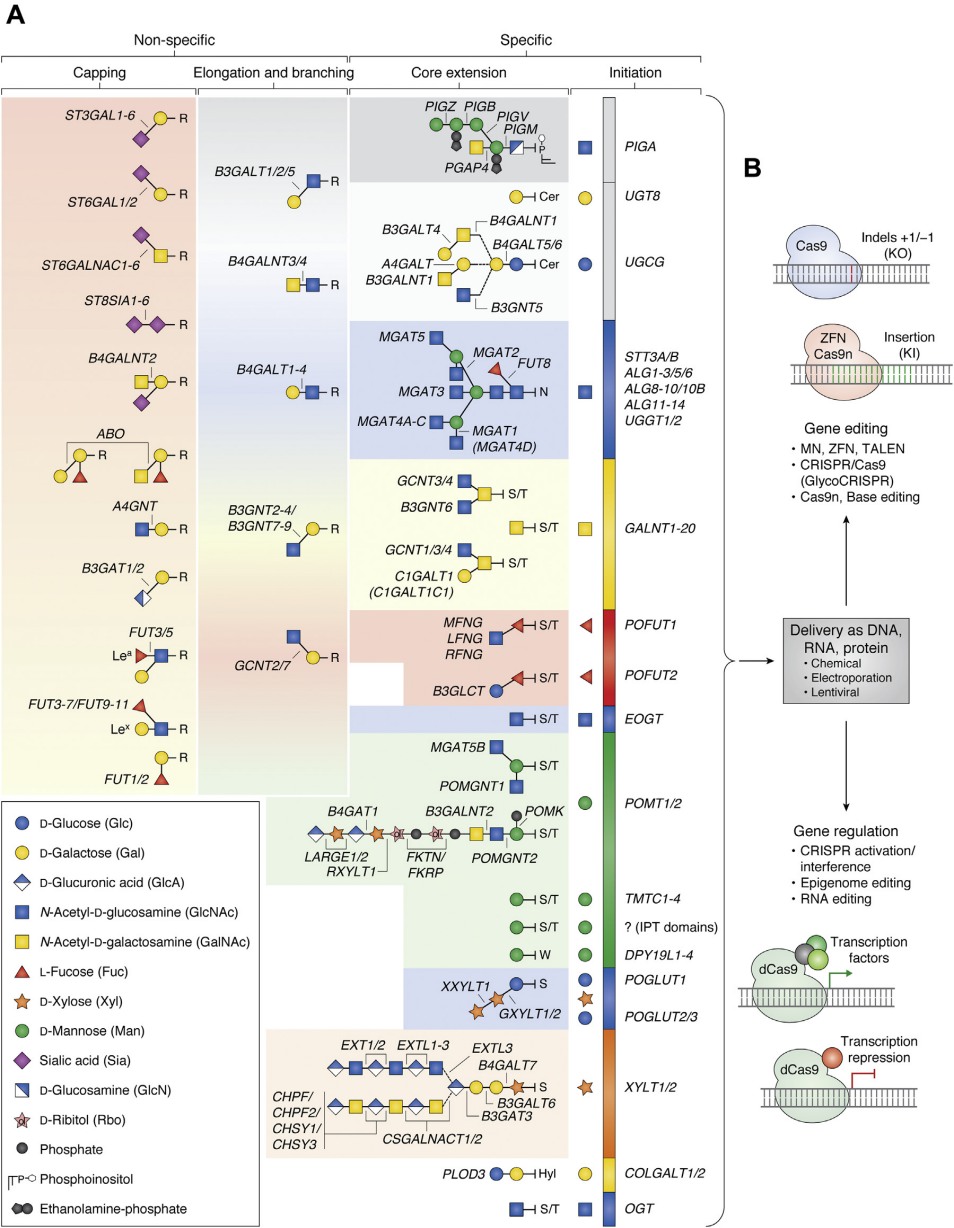
**Overview of principles and strategies for stable genetic engineering of cellular glycosylation capacities.***A*, overview of the 16 human glycosylation pathways with predicted assignments of 173 glycosyltransferase genes to the major biosynthetic steps using the rainbow display organization (5, 199). The rainbow depiction of glycosylation pathways illustrates the major biosynthetic steps organized into pathway-specific steps (*right part* with even colored according to the initial monosaccharide except for glycolipids) and pathway nonspecific steps (*left part* with toned colors) with predicted GT genes assigned. Note that this is a simplified scheme of pathways and GT genes are assigned only to the primary predicted functions. Genetic engineering of glycosylation requires considering the properties of individual enzymes and their potential effects on the cellular glycosylation pathways. Loss or gain of a GT may have highly specific effects or wider effects on multiple glycosylation pathways. Glycosylation steps covered exclusively by one unique enzyme (nonredundant steps), *e.g.*, core α6-fucosylation of N-glycans by FUT8, yield highly specific and predictable outcomes with loss/gain engineering. Steps covered by multiple isoenzymes with overlapping functions (redundant steps) may or may not yield easily predictable outcomes, and the outcome may vary in cells dependent on the expression of such isoenzymes. Most steps in elongation and branching and capping are covered by partial redundancies by multiple isoenzymes. For example, sialylation by any of the four sialyltransferase subfamilies is covered by partial redundancies, and, *e.g.*, combinatorial KO of three genes is required to selectively eliminate α3-sialylation on N-glycans (KO of *ST3GAL3/4/6*), while KO of two genes is required to selectively eliminate α3-sialylation of core1 O-glycans (KO *ST3GAL1/2*). Glycan symbols are displayed in the Symbol Nomenclature for Glycans (SNFG) format (340). *B*, graphic depiction of current nuclease-based gene-editing tools for knockout (KO) and knock-in (KI) of genes, and emerging CRISPR-based technologies for regulating and activating gene expression.

The glycosylation machinery of a given cell determines the ensemble of glycan structures and types of glycoconjugates that constitute the glycome of that same cell (31). The glycosylation capacities of different cell types vary primarily through changes in expression of the GTs that directs different steps in glycosylation pathways (Fig. 2*A*); however, a number of other factors affect the glycosylation outcome (5, 32). GTs have different donor sugar and acceptor substrate specificities often with considerable overlaps (33), and glycosylation reactions are primarily guided by the kinetic properties of the expressed ensemble of enzymes (2, 34, 35). Glycosylation is therefore akin to a large integrated metabolic network involving a myriad of enzyme reactions, where a number of “rules” can be applied to predict the outcomes in terms of glycans being produced. While many of these rules for enzyme reactions can be applied with a high degree of fidelity, others involve less predictable outcomes. Thus, rational engineering of glycosylation in cells requires information on glycosylation processes and often experimentation and validation of outcomes.

Figure 2*A* organizes glycosylation into 16 distinct pathways and major biosynthetic steps with assignment of predicted roles of 173 human GTs (note that we refer to GTs by their gene names only for simplicity; capital letters refer to human GTs, and italics refer to genes) (5, 34, 36). The map provides a blueprint for rational engineering of glycosylation in mammalian cells and highlight: i) GTs that are unique and serve glycosylation pathway-specific roles (essentially all steps in initiation of glycosylation on lipids and proteins and most early steps in core extension) and GTs that serve pathway nonspecific roles (assembly of elongated and branched glycans and the final capping); and ii) glycosylation steps that are covered by single nonredundant GTs and those that are covered by partial redundancies from GT isoenzymes. Moreover, the map serves to translate cellular expression data for GTs from, *e.g.*, single-cell transcriptomics or proteomics into predicted glycosylation capacities and glycome outcomes, as recently demonstrated (34). The glycosylation capacities of immortalized mammalian cell lines such as the CHO and HEK293 cells widely used for recombinant expression of glycoproteins can be extensively manipulated by multiple KO/KI events without major effects on viability in simple cultures and performance in production and secretion (34). The only essential steps for cell viability may be complete loss of the function of the oligosaccharyltransferase (OST) complex and initiation of N-glycosylation and complete loss of the cytosolic O-GlcNAcylation orchestrated by the protein O-GlcNAc transferase (OGT) (37, 38).

A major incentive for using genetic engineering of glycosylation in studying the biology of the glycome is that this is one strategy that allows probing the glycome at the single cell level. Engineering glycosylation capacities may be used to explore biological functions of glycosylation in a cell by dissection of the glycosylation genes and enzymes required for the particular function. Knowledge of the glycosylation pathways in the cell may then be used to predict the structural features of the glycome that underlies the function. Emerging high-quality single-cell transcriptomics and proteomics data can also be used to gauge the expression of glycosylation enzymes and with some confidence used to predict the cellular glycosylation capacities and glycome outcome (5). This information may be incorporated in engineering strategies to simplify and focus the design of genetic engineering on relevant active glycosylation pathways. Currently, analytic glycome and glycomics technologies are not at this level of sensitivity, and structural analytic data sets are generally sums of glycome contributions from multiple cells often with heterogeneous origins (39, 40). The glycome of any cell at a given state and timepoint is predicted to be less heterogeneous than current analytics suggest, and distinct glycosylation features may be highly regulated (35, 41, 42, 43). Decades of studies with glycan-binding proteins (GBPs) including lectins and antibodies have shown that the expression and distribution of a limited number of glycan epitopes or structural glycan features change characteristically during cellular maturation and differentiation, tissue formation, and in diseases (44, 45, 46, 47, 48). Despite progress though, we are still limited in our abilities to decipher the molecular basis of specific roles of glycosylation in biological functions, and genetic glycoengineering in mammalian cells is one promising strategy for discovery and dissection.

## The tools—precise genome-editing techniques

As pointed out above, genetic engineering of glycosylation in mammalian cells has been performed for decades. Considerable efforts have been devoted to overexpression and/or silencing of GTs (and other metabolic enzymes, transporters, hydrolases) in mammalian cells (49, 50, 51, 52, 53, 54). However, overexpression of GTs may lead to markedly perturbed glycosylation (55), and gene silencing using RNA interference strategies often results in limited and variable reduction of enzyme levels with uncertain effects on glycosylation outcomes (4, 56, 57). Today, glycoengineering with precision genome-editing techniques overcomes these issues and enables stable genetic changes to the cellular glycosylation machinery. The rapidly expanding toolbox for precise genome editing offers wide opportunities for targeted KO, KI, or modulation of the expression of glycogenes to reprogram glycosylation in mammalian cells. In the following, we briefly review these tools and refer to original studies for details (58, 59, 60, 61, 62, 63, 64).

### Targeted KO

Stable gene KO can be achieved using nuclease-based genome engineering particularly ZFNs, TALENs, and CRISPR/Cas that all can be designed to bind and cleave specific nucleotide sequences and introduce double-strand breaks (DSBs) (65, 66). Repair of DSBs through nonhomologous end joining (NHEJ) is error-prone and results in mutations or small insertions/deletions (indels) that disrupt the target gene (67) (Fig. 2*B*). ZFNs and TALENs have very high sequence binding specificity and low off-target effects (68); however, design options for target sites are limited and production is laborious, costly and requires expertise although premade targeting constructs are available. CRISPR/Cas gene editing only requires design of guide RNAs (gRNAs), synthetic RNAs with a 16 to 20 nucleotide genomic targeting sequence (crRNA) with a proto-spacer adjacent motif (PAM), and a Cas nuclease-recruiting sequence (tracrRNA) (58). Different Cas nucleases exist in different species each recognizing distinct PAMs, and currently this specificity is further engineered to allow broader sequence recognition (69). Most CRISPR engineering is based on the class II nucleases Cas9 from *S. aureus*, to which we refer if not otherwise stated, and more recently Cas12. Established prediction algorithms for optimal candidate gRNA designs are continuously developed; however, candidates still need experimental validation for functional activity (70, 71, 72, 73). Libraries of gRNAs for specific targeting of all human glycosyltransferase genes are now available, including a library of validated high efficiency single gRNAs (GlycoCRISPR) (74) and a lentiviral viral library containing ten gRNAs (predicted design) per target gene (75). Targeted gene engineering is greatly facilitated by the Indel Detection by Amplicon Analysis assay, which provides single base resolution and informs of the spectrum and frequencies of indels enabling screening for and selecting indels resulting in frameshifts and functional KOs (76, 77). Detection of indels may also be based on enzymatic mismatch cleavage assays (78, 79) and of course different sequencing strategies (80).

### Targeted KI

Site-directed and stable introduction of target genes can be achieved by nuclease-based KI. In the presence of a homologous DNA template containing target gene of interest, DSBs can be repaired *via* homology-directed repair (HDR) resulting in the genomic integration (Fig. 2*B*). However, HDR occurs at a lower frequency and only in the S/G2 phase compared with the dominant NHEJ repair pathway operative throughout the cell cycle (mainly G1 phase), which makes KI *via* HDR more challenging (67). Therefore, several strategies apply KI independent of the HDR pathway. For example, the obligate ligation-gated recombination (ObLiGaRe) strategy utilizes ZFNs or TALENs to create complementary DNA overhangs (sticky ends) in both the genomic target site and the donor DNA allowing its integration (60, 81). This KI strategy has already been successfully used for stable KI of GTs (34, 82).

CRISPR/Cas9-mediated KI utilizes the microhomology-mediated end joining (MMEJ) pathway to repair DSBs (with blunt ends), and foreign DNA flanked with short homologous sequences (5–25 bp) can be used for integration at target sites (83, 84, 85). CRISPR strategies have improved KI efficiency *via* HDR by virtue of Cas9 nickases (Cas9n), Cas9 nucleases with an inactivating mutation in either the HNH (Cas9H840A) or RuvC (Cas9D10A) domain (59, 62, 86). Cas9n creates single-strand breaks (nicks), and by pairing two nickases and two gRNAs for both DNA strands, DSBs with long overhangs can be created. Donor DNA flanked with complementary arms or simultaneously nicked donor DNA can then be inserted at the DSB site *via* HDR or non-HDR mechanisms with high efficiency and low off-target effects (62, 87, 88, 89). To overcome random integration of artificial DNA sequences into the genome and off-target effects, the AAVS1 locus is often used as safe-harbor site for KI (61, 90). Gene KI can make use of customizable constructs for constitutively active expression of target genes, for example, with or without tags and also holds the opportunity to insert tunable expression constructs, for example, under control of the Tet-On/Off system, and this has already been applied for glycoengineering (91).

### Regulating endogenous gene expression

The CRISPR technology is rapidly evolving by adding functionalities to Cas nucleases. Utilization of catalytically inactive (dead) dCas9 allows to fully repress target gene expression through fusion with transcription repressors (CRISPR interference, CRISPRi) and recruitment to the promoter region of a target gene (92). Likewise, fusion with transcriptional activators such as VP64, p65, and Rta enables activation of gene expression (CRISPR activation, CRISPRa) (63, 64, 93) (Fig. 2*B*). CRISPRa was applied to activate the fucosyltransferase 4 and 9 genes (*fut4* and *fut9*) in a murine cell line resulting in Lewis^x^ expression (94). Other CRISPR-based approaches for gene regulation have emerged that are potentially useful for glycoengineering. For example, dCas9 fused to base editors such as cytidine deaminase that coverts cytidine to uridine allows introduction of targeted point mutations (base editing), fusion to a reverse transcriptase domain for prime editing, fusion to epigenetic modifiers (*e.g.*, P300, TET1, LSD1, DNMT3A) enables epigenome editing, RNA-targeting Cas13a nuclease enables posttranscriptional engineering of mRNA, and CRISPR tools are emerging for mutagenesis and directed evolution (27, 80, 95, 96, 97). Currently, the main obstacle for extensive glycoengineering is limitations in introducing multiple genes by targeted KI, and this obstacle may be alleviated by activation of endogenous nonexpressed genes. Another interesting opportunity for engineering is to tag endogenous GTs or their substrate proteins to enable selective monitoring of expression of enzymes or their effects on specific proteins in live cells (98).

### Delivery gene editing

A key step in genetic engineering is the delivery of editing components such as DNA, RNA, and/or protein (99, 100) (Fig. 2*B*). DNA plasmids are most commonly used, because of ease of availability and use, stability, and flexibility in design, but genomic insertions of plasmids may lead to off-target effects. Use of RNA circumvents this as RNA is readily translated in the cytoplasm resulting in fast protein production, and transfecting with RNA has advantages especially with primary and difficult-to-transfect cells (101). However, RNA is more costly and difficult to handle, unstable, less customizable, and expression of the encoded protein is lower and relatively short-lived. The Cas9 protein may be delivered precomplexed with gRNAs and used directly in transfection allowing for control over dosage, and this may be effective with cells difficult to transfect with DNA or RNA, but requires highly pure protein and protocol optimization for different proteins. The methods of delivery include chemical, biological, or physical means, and the efficacy of these varies greatly (27, 99, 100). For example, the human embryonic kidney (HEK293) cell is readily transfectable with cost-effective chemical reagents such as calcium phosphate, polyethylenimine (PEI) polymers, and liposomes. More difficult-to-transfect cells such as primary cells and nondividing cells may be efficiently transfected by electroporation, nucleofection, or other methods that temporarily induce pores in the plasma membrane; however, these often result in varying levels of cytotoxicity (27, 102). Lentiviral transduction may be the option of choice for difficult-to-transfect cells and enables stable integration of highly customizable vectors. This was applied, *e.g.*, for glycoengineering of human keratinocytes to develop organotypic skin models (103), and a comprehensive lentiviral glycogene CRISPR library has been designed (75). Notably, level and timing of Cas9 expression are difficult to control with these methods and high prolonged expression enhances off-target frequencies (77). This hurdle may be overcome by inactivating CRISPR systems that allow controlled and temporal expression levels of Cas9, and these have already been applied to reduce off-target effects of editing the human α3-sialyltransferase 4 gene (*ST3GAL4*) (104). Selection markers such as antibiotics or fluorescent reporters, *e.g.*, GFP-tagged Cas9, facilitate enrichment of transfected cells with desired nuclease expression levels. Fluorescence activated cell sorting (FACS) is useful to select and enrich for cells with desirable expression of GFP-tagged Cas9 (77, 105), as is further selection of clones with desirable changes in glycosylation and/or expression of targeted proteins by lectins and antibodies.

## Engineering of glycosylation capacities in cells

Two conceptionally opposing strategies for glycoengineering have been applied. One approach takes origin in glycan structures and seeks to identify genes required for biosynthesis and expression of specific glycans—here designated as random glycoengineering. The other approach takes origin in the glycosylation capacities of a cell and seeks to engineer the repertoire of expressed GTs to accommodate biosynthesis of glycans of interest—here designated as rational glycoengineering. These strategies are not mutually exclusive.

### Random glycoengineering—discovery potential

Random glycoengineering was pioneered by Stanley *et al.* who generated glycosylation mutants in Chinese hamster ovary (CHO) cells, designated Lec or LEC (for loss-of-function and gain-of-function mutants, respectively). CHO cells were subjected to random mutagenesis followed by positive or negative selection with cytotoxic plant or bacterial lectins to obtain lectin-resistant mutants with loss/gain of a glycan feature. With this approach a large number of valuable CHO glycosylation mutants were isolated leading to the discovery and characterization of GTs, sugar nucleotide transporters, and other genes (10, 106). Later, more systematic studies with random insertional mutagenesis using gene trapping in haploid HAP1 cells and selection with Lassa virus binding to the matriglycan on α-dystroglycan revealed several known (*e.g.*, *POMT1/2*, *B3GNT1*) and unknown (*e.g.*, *SLC35A1*, *ST3GAL4*, *B3GALNT2*) glycogenes involved in glycosylation of α-dystroglycan and underlying dystroglycanopathies (107). Random mutagenesis studies are now being substituted by whole genome screens (or select gene subsets) using CRISPR libraries (108, 109). These and other studies have uncovered unexpected genes that direct or regulate cellular glycosylation processes in interactions with bacterial toxins, viral infections, and other molecular events as summarized in Table 1. This strategy relies on the efficiency of CRISPR/Cas targeting in introducing biallelic indels (and multiallelic) in target genes necessary to induce complete loss of function of GT enzyme activities and thus partly omitting the benefits of using haploid cell lines where mainly a single allele of genes requires targeting. Technical limitations of genome-wide CRISPR/Cas KO screens are off-target effects and false-negative hits caused by nondeleterious indels (108, 109), but also a low likelihood of identifying GTs that are members of partially redundant isoenzyme families (5). Thus, the CRISPR/Cas screening strategy has mainly identified nonredundant genes with global effects on glycosylation (Table 1). For example, genome-wide screening for genes required for avian influenza virus replication identified the Golgi CMP–sialic acid transporter (*SLC35A1*) and confirming requirement for sialic acid receptors; however, none of the many sialyltransferase genes necessary for sialylation were identified (110). A genome-wide CRISPR screen for hepatitis A virus entry identified multiple genes in the ganglioside biosynthetic pathway, including UDP-glucose ceramide glucosyltransferase (*UGCG*) and β4-galactosyltransferase 5 (*B4GALT5*) directing synthesis of Galβ1-4Glcβ1-Cer, and the lactosylceramide α3-sialyltransferase (*ST3GAL5*), which may only serve as the GM3 synthase (NeuAcα2-3Galβ1-4Glcβ1-Cer) (111). Multiple genome-wide screens have shown the importance of N-glycosylation and the OST complex for Dengue virus infection (112, 113, 114), and one screen in the HAP1 haploid cell line also identified multiple genes in the heparan sulfate pathway indicating a role of GAGs as well (115).

**Table 1:** Genome-wide screens in mammalian cells identifying genes involved in glycosylation

Cell type	Genome-wide screen	Phenotypic selection	Identified glycogenes	Reference
Chronic myeloid leukemia cells HAP1 (haploid)	Gene trap mutagenesis	Resistance to Lassa virus entry *via* α-dystroglycan	*POMT1*/*2*, *B3GNT1*, *SLC35A1*, *ST3GAL4*, *B3GALNT2*	(107)
Human cervical cancer cells HeLa	CRISPR/Cas9 KO screen^a^	Resistance to Shiga toxin (Stx)	*UGCG*, *B4GALT5*, *SPTLC2*, *A4GALT*, *SPTLC1*	(328)
Human cervical cancer cells HeLa	CRISPR/Cas9 KO screen^a^	Resistance to Shiga toxin (Stx)	*A4GALT*, *B4GALT5*, *UGCG*, *GALE*, *SLC35A2*	(329)
Human bladder cancer cells 5637	CRISPR/Cas9 KO screen^a^	Resistance to Shiga-like toxins (Stxs) 1 and 2	*A4GALT*, *B4GALT5*, *SLC35A2*, *UGCG*, *SLC35A2*	(330)
Human colorectal adenocarcinoma cells HT-29	CRISPR/Cas9 KO screen^b^	Resistance to EHEC cytotoxicity (T3SS, Stx1 and Stx2)	*A4GALT*, *B4GALT5*, *UGCG*	(331)
Human cervical cancer cells HeLa	CRISPR/Cas9 KO screen^a^	Resistance to ricin toxin	*ALG5*, *ALG6*, *ALG8*, *MOGS*, *OST4*, *MAN1A2*, *MAN2A1*, *MGAT1*, *MGAT2*, *SLC35C1*, *FUT4*	(330)
Human cervical cancer cells HeLa	CRISPR/Cas9 KO screen^a^	Resistance to *E. coli* subtilase cytotoxin (SubAB)-induced cell death	*SLC39A9*, *CMAS*, *SLC35A1*, *MGAT1*, *C1GALT1*, *C1GALT1C1*	(332)
Human cervical cancer cells HeLa	CRISPR/Cas9 KO screen^a^	Gal-3 cell surface localization	*SLC35A2*, *MGAT1*, *MAN1A2*, *SLC39A9*	(129)
Human hepatocellular carcinoma cells Huh7.5.1	CRISPR/Cas9 KO screen^a^	Resistance to Ebola virus infection	*SLC30A1*, *GNPTAB*	(333)
Human cervical cancer cells HeLa and human embryonic kidney cells HEK293	CRISPR/Cas9 KO screen^a^	Resistance to West Nile virus infection	*STT3A*, *OST4*, *OSTC*	(334)
Chronic myeloid leukemia cells HAP1 (haploid)	CRISPR/Cas9 KO screen^a^	Resistance to Dengue virus infection	*STT3A*, *STT3B*, *RPN2*, *B4GALT7*, *B3GALT6*, *B3GAT3*, *PAPSS1*, *SLC35B2* *DPM1*, *DPM3*	(115)
Chronic myeloid leukemia cells HAP1 (haploid) and Human hepatocellular carcinoma cells Huh7.5.1	CRISPR/Cas9 KO screen^a^	Resistance to Dengue virus infection	*STT3A*, *STT3B*, *MGAT1*, *OSTC*, *RPN1*,*RPN2*, *OST4*, *B3GALT6*, *EXT1*	(113)
Human lung epithelial cells A549	CRISPR/Cas9 KO screen^a^	Resistance to IAV (H5N1) virus infection	*SLC35A1*, *DPM2*, *ALG3*, *ALG4*, *ALG12*, *GANAB*, *A4GALT*, *B3GAT1*, *B4GALNT4*, *CHSY1*, *CSGALNACT2*, *HS3ST6*, *PIGN*, *DPM2*	(110)
Human lung epithelial cells A549	CRISPR/dCas9 activation screen^c^	Blocking IAV (H1N1/PR8/1934) infection	*B4GALNT2*	(116)
Human colorectal adenocarcinoma cells HT-29	CRISPR/Cas9 KO screen^b^	Resistance to *V. parahaemolyticus* cytotoxicity (T3SS1 and T3SS2)	*SLC35B2*, *SLC35B3*, *HS6ST1*, *SLC35C1*, *GMD*, *FUT4*, *SLC35A2*	(335)
Human lymphoma cells JeKo-1	CRISPR/dCas9 activation screen^c^	Resistance to anti/CD3xCD20 bispecific antibody-mediated killing	*B4GALNT1*, *B3GNT4*	(336)

a Lentiviral GeCKO sgRNA library targeting 19,050 genes and 1864 miRNAs.

b Lentiviral AVANA sgRNA library targeting 18,675 genes.

c Lentiviral sgRNA library targeting upstream TSS of 23,430 coding isoforms.

Genes that are not endogenously expressed in the cell line used for screening are of course disregarded, but this limitation may partly be overcome with the emerging genome-wide CRISPR activation libraries that enable gain-of-function screening. For example, a genome-wide CRISPR/dCas9 screen for inhibitory factors of α2-3Sia binding influenza A virus uncovered β4-GalNAc-transferase 2 (*B4GALNT2*) and revealed that addition of a β4GalNAc residue to α2-3Sia capped glycans to form the SDa epitope (Neu5Acα2-3[GalNAcβ1-4]Galβ1-R) blocks binding (116). The unbiased nature of random engineering studies has demonstrated great potential for discoveries of GT genes and their functions in cellular glycosylation processes.

### Rational genetic glycoengineering—custom design and dissection

Rational glycoengineering represents a systematic and versatile strategy for studying and dissecting biological roles of glycosylation. Advanced understanding of the genetic and biosynthetic circuits of the cellular glycosylation machinery and regulation of the glycome (Fig. 2*A*) (2, 5, 117, 118), combined with the facile gene-editing technologies and libraries of validated gRNAs such as the GlycoCRISPR resource (74), is enabling wide use of rational glycoengineering by KO/KI of genes. There appears to be few restrictions to the extent of reprogramming of glycosylation possible in cell lines, and extensive engineering of a majority of the known GT genes has been performed in mammalian cells (predominantly CHO and HEK293) (34, 82, 103, 119, 120, 121, 122, 123). Thus, it is now essentially possible to consider the blueprint of glycosylation pathways (Fig. 2*A*) and deconstruct and/or reconstruct any of these without substantially affecting cell viability and performance, or, for example, expression and secretion of recombinant glycoproteins (121). Rational genetic engineering may also apply to the many enzymatic modifications of glycans including epimerization and attachment of sulfate, phosphate, acetyl, methyl, and other groups (31). The glycome is further shaped by endogenous glycoside hydrolases and in particularly the four mammalian neuraminidases (NEU1–4) may affect the degree of sialic acid capping (124). Design of rational glycoengineering experiments needs to consider the enzymes expressed in the cell of choice and potential genetic redundancy for biosynthetic steps provided by isoenzymes. Single-cell RNAseq transcriptomics (and to some extend proteomics) may provide information on the repertoire of expressed GTs and other relevant enzymes, and this may be useful for the engineering design considering the current knowledge of glycosylation pathways in cells (Fig. 2*A*). However, it is important to acknowledge that interpretation of such data still requires caution. Further advances are needed to be able to reliably predict glycosylation outcomes based on quantitative levels of enzymes in cells, and the predicted roles of GTs in the different glycosylation pathways and biosynthetic steps need validation.

Targeting biosynthetic steps in glycosylation that are controlled nonredundantly by a single unique GT generally results in predictable global outcomes. For example, KO of *FUT8* that solely directs transfer of α1-6 fucose to the innermost GlcNAc residue of the chitobiose core of N-glycans is sufficient to eliminate this glycosylation feature on N-glycoproteins (24, 25, 125). Many steps in the initiation and immediate core extension of glycosylation pathways are controlled by nonredundant enzymes (Fig. 2*A*), and this is used to dissect roles of elaborated glycans on different types of glycoconjugates by targeting the earliest committed biosynthetic steps in core extension. Thus, KO of the Glc-Cer β4-galactosyltransferase *B4GALT5*(*6*) gene(s) eliminates synthesis of elaborated glycolipids, KO of the α3-mannosyl-glycoprotein β2-GlcNAc-transferase *MGAT1* gene eliminates elaboration of N-glycans, and KO of the core1 synthase *C1GALT1* gene or the private chaperone COSMC eliminates elaboration of the most common types of O-glycan (34, 103, 126, 127, 128, 129). Rational engineering may lead to discoveries and challenge the current understanding of genetic and biosynthetic regulation of glycosylation pathways. For example, when the—at the time—known genes controlling protein O-mannosylation (*POMT1/2*) were KO in mammalian cells followed by analysis of the O-Man glycoproteome, two new O-mannosylation pathways directed by previously unknown enzymes were discovered (130, 131). Another example of glycoengineering leading to discoveries involved the use of forward genetic screening to identify the two enzymes directing the Galβ1-3GalNAcβ1-4 branch attached to the first Man residue of the glycosylphosphatidylinositol (GPI)-anchor (132). The post-GPI attachment to proteins GalNAc-transferase 4 (PGAP4) predicted to attach the first β4GalNAc was validated by KO of the *PGAP4* gene, and a genome-wide CRISPR/Cas screen identified the β3-galactosyltransferase 4 (B3GALT4) as the enzyme extending the β4GalNAc (133). B3GALT4 also functions as the GM1 glycolipid synthase (134, 135). A cell model was first engineered by KO of GPI transamidase component PIG-S gene (*PIGS*), encoding a subunit of the GPI-Tase that transfers GPI to proteins, to block protein transfer of GPIs and enable screening for loss of galactosylation by an antibody detecting the Galβ1-3GalNAcβ epitope on free GPIs. Interestingly, in the process of dissecting the putative dual role of B3GALT4, it was discovered that UGCG that initiates glycolipid biosynthesis is required for B3GALT4 functions in both glycolipid and GPI biosynthesis. These studies thus not only uncovered an unexpected cross talk between two different glycosylation pathways, but also led to discovery of interaction between UGCG and B3GALT4. The Ribitol β4-xylosyltransferase (TMEM5) required for biosynthesis of the core O-mannosyl matriglycan of dystroglycan was originally identified in a HAP1 screen and validated by KO showing that the Xylβ1-4Rbo5P structure was disrupted (136).

A “brutal” variant of rational glycoengineering is to target genes that serve glycosylation in global ways such as in synthesis and transport of nucleotide sugar donors, *e.g.*, as demonstrated already with the CHO Lec mutants (10). KO of *SLC35A1* results in loss of all types of sialylation (137, 138). The original discovery that loss of the UDP-Glc/GlcNAc C4-epimerase (GALE) in the CHO ldld cell model resulted in deficiencies of UDP-Gal/GalNAc and impaired glycosylation of N- and O-glycoproteins, a defect that can be reversed by exogenous addition of Gal and/or GalNAc sugars (139, 140), is now being replicated in other cells by targeted KO of *GALE*/*GALK1*/*2* to install the unique ability to regulate glycosylation by exogenous addition of sugars (141, 142). Similarly, KO of the isoprenoid synthase domain-containing protein ISPD required for synthesis of CDP-Ribitol blocks synthesis of the matriglycan on α-dystroglycan (143, 144).

Targeting biosynthetic steps that are covered by partial redundancy from isoenzymes remains a challenge for predicting outcomes of rational glycoengineering. However, targeting isoenzymes also present unique opportunities to uncover their nonredundant functions. For isoenzymes that function in pathway specific steps, such as the many polypeptide GalNAc-transferases (GALNTs), selective KO/KI of individual isoenzyme genes (*GALNT1-20*) in cell models was very useful to dissect non-redundant functions using differential O-glycoproteomics (145, 146, 147). This strategy provides deeper and more unbiased insights into substrates for GALNTs compared with *in vitro* enzyme assays with short peptides and has revealed important isoform-specific targets such as the O-glycosylation of the ligand-binding region of the low density lipoprotein receptor-related receptors directed exclusively by GALNT11 (148, 149, 150). A particularly useful extension of this strategy is to install inducible expression of GT isoenzymes to explore their functions in regulating glycosylation. Thus, engineering cells with KO of an endogenous *GALNT* and reinstallation of the same isoenzyme under stringent inducible expression by KI enabled the remarkable observation that increasing levels of the GALNT isoenzyme produce tight regulation of nonredundant substrates without interference or change to the majority of redundant substrates (91). KO screen of *GALNT*s was used to show that GALNT1 plays an essential role in the glycosylation of the mucin domain of the Ebola surface glycoprotein and the function of this in cell detachment and hemorrhaging (151). Engineering *GALNT* genes in a human keratinocyte skin model showed that distinct isoforms serve unique functions in epithelial formation and differentiation (103). Similarly, a characteristic *de novo* expression of the GALNT6 isoenzyme in colon cancer was investigated by engineering *GALNT*s in a colon cancer cell line, and *GALNT6* was selectively shown to affect differentiation and growth (152). The unique functions of several other GALNT isoenzymes were also explored in KO studies (153, 154, 155, 156, 157, 158, 159). Moreover, glycoengineering is being used with approaches for metabolic labeling and tagging of glycosylation (22, 23, 160, 161, 162), including the azido sugars for click chemistry introduced by the Bertozzi group (163, 164, 165). Glycoengineered cells allow for screening and validation of selective binding and labeling probes in live cells as exemplified by the bump-and-hole strategy employed by Schumann and colleagues (166), in which modified UDP-GalNAc donor substrates (bumped) and GALNTs engineered to selectively accommodate these by an enlarged active site (hole) are used to detect isoform-specific functions in transfected cells (167, 168, 169).

In N-glycosylation the functions of the two dolichyl-diphosphooligosaccharide protein GT STT3A and STT3B subunits of the heteromeric OST complex were analyzed by proteomics studies of HEK293 cells with KO of either subunit, demonstrating distinct modification sites for the STT3A/B subunits (170, 171). Similarly, KO targeting was used to demonstrate that both *FKTN* and *FKRP* are Ribitol 5-phosphate transferases (172), that both procollagen galactosyltransferase 1 (COLGALT1) and 2 (COLGALT2) function in galactosylation of hydroxylysine (HYL) in collagens (173, 174), that both protein O-glucosyltransferase 2 (POGLUT2) and 3 (POGLUT3) serve the same Notch EGF11 repeat sequence (C^3^-XNTXGSFX-C^4^) different from POGLUT1 (175), and that the substrate specificities of some of the four DPY19L1-4 C-mannosyltransferases may differ with respect to Trp substrate sites in consecutive glycosylation site motifs (*e.g.*, the WXXWXXWXXC sequence in thrombospondin repeats found in the netrin receptor UNC5A) (176).

However, most isoenzymes function in glycosylation pathway nonspecific steps with considerable cross talk between pathways, and these isoenzymes direct terminal structural features that determine many of the biological interactions with, *e.g.*, GBPs (Fig. 2*A*). This includes the enzymes assembling the elongation and branching of LacNAc disaccharides (Galβ1-3/4GlcNAcβ1-R) and LacDiNAc (GalNAcβ1-4GlcNAcβ1-R) termini and the capping steps by the many sialyltransferase and fucosyltransferase isoenzymes (177, 178, 179, 180, 181, 182, 183, 184). Since these glycan structures are found widely on N-glycans, O-glycans, and glycolipids, glycoengineering may affect different types of glycoconjugates and complicate analysis and assignments of functions. Redundant functions of the B4GALTs (B4GALT1-4) in galactosylation of N-glycans have been shown by combinatorial KO in CHO cells (82, 185). The many sialyltransferase isoenzymes were explored by KO revealing that ST3GAL4, and not ST3GAL3/6, is the major contributor in forming the sialyl-Lewis^X^ (SLe^x^) glycan epitope that mediates selectin binding to lymphocytes and directs lymphocyte trafficking and extravasation (186). Glycoengineering was also used to demonstrate that enterovirus D68 infection requires both α2-3Sia and α2-6Sia recognition (187) and that human noroviruses require the blood group H glycan epitope directed by the secretor α2-fucosyltransferase (FUT2) (188). More emphasis on the nonredundant functions of isoenzymes is clearly needed as these most likely provide for differential regulation of important functions (5, 118). Combinatorial engineering of such isoenzymes can be used to weed out specific nonredundant functions with appropriate assays as discussed above (34).

## Novel opportunities provided by nuclease-based glycoengineering

In the following we highlight examples for how precision engineering of glycosylation is providing new opportunities for basic and translational sciences.

### Cell-based glycan arrays

**Figure 3:**
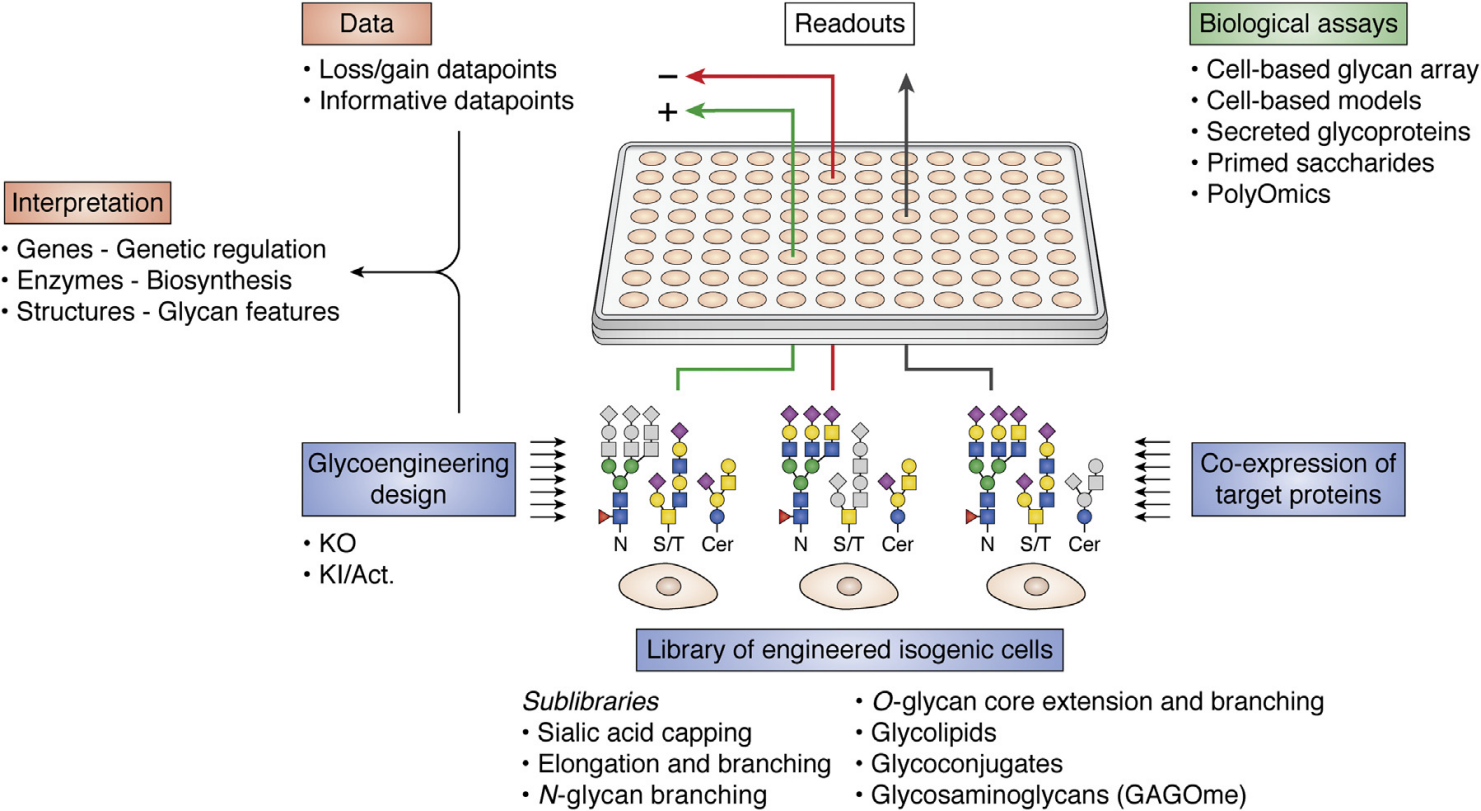
**Depiction of concepts of cell-based glycan arrays.** KO/KI (or activation) of GT genes in a mammalian cell line are used to generate isogenic cell lines with loss/gain of select glycosylation capacities and hence display of loss/gain of select glycan features on endogenous surface glycoconjugates. Libraries of such isogenic cell lines constitute the cell-based glycan arrays (34), and sublibraries can be designed to dissect select glycosylation steps or glycosylation pathways by combinatorial engineering of GTs (and other genes such as sulfotransferase genes modifying glycans). The design of libraries may be guided by the glycosylation pathways outlined in Figure 2*A* and take into account the endogenous GTs expressed in the cell. The cell line of choice, *e.g.*, HEK293, may not express protein substrates of interest and coexpression (or activation of the endogenous gene) of one protein (or groups of) can therefore be included. The cell-based array can be probed by any biological assay suitable with live or fixed cells, but usage requires a positive signal. The positive signal may, for example, be found with the wildtype cells, induced during library screening by loss/gain of glycosylation features, or established from the start by, *e.g.*, introducing a protein not endogenously expressed. The readouts are loss (*red arrow*)/gain (*green arrow*) of signals or data points (*e.g.*, of binding to cells), and most datapoints will be neutral, *i.e.*, no change in binding (*gray arrow*). Some neutral datapoints may still be informative in narrowing down structural features involved or not involved in interactions, especially when the genes affected are predicted to function in the same glycosylation pathway as those providing loss/gain of signals. Interpretation of the collected datapoints is performed with reference to the outlined glycosylation pathways and gene engineering matrix employed. The primary result being the GT genes required for signal (with added support from GTs genes not required), the secondary result being the biosynthetic pathway(s) and enzyme(s) required (and not), and the final result being the predicted glycan features and structures involved. This is thus different from studies with traditional glycan arrays that directly report glycan features and structures involved in binding to defined glycans.

Glycan arrays have had huge impact on our insight into protein interactions with glycans and dissecting structural glycan features directing binding specificities of GBPs (189, 190, 191, 192, 193, 194). Current glycan arrays generally comprise printed oligosaccharides, and these arrays are highly useful for dissecting structural features of glycans involved in binding of GBPs. Printed arrays with glycolipids and glycopeptides arrays or lipids are also being developed (195, 196, 197, 198). A major advantage of printed glycan arrays is that these enable analysis of binding to distinct compounds with defined structures; however, they may not present glycans in the natural context of glycoconjugates and the cell surface. These drawbacks may at least partly be met by cell-based glycan arrays comprised of libraries of isogenic cells genetically engineered to display distinct features of the glycome (34) (Fig. 3). Mutant cells with global deficiencies in glycosylation features such as sialylation or glycosaminoglycan biosynthesis have been widely used in the past (10, 106). It is now possible to develop libraries of stable isogenic cells that individually display the cellular glycome with loss or gain of distinct glycosylation features by systematic and combinatorial KO/KI of GT genes, and these libraries can constitute comprehensive cell-based glycan arrays. A dedicated cell-based array for glycosaminoglycans was developed in CHO cells—designated the GAGOme (120), and another library for HS GAGs in mouse lung endothelial cells was generated by a combination of immortalizing cells from KO mice and CRISPR/Cas gene editing (123). A more comprehensive array for the human glycome covering many features for glycolipids, N-glycoproteins, and GalNAc-type O-glycans was developed in HEK293 cells (34, 199).

Cell-based glycan arrays primarily inform of the glycosylation genes and biosynthetic steps involved in a queried GBP or biological function, and this information is used to predict the structural features of glycans involved (Fig. 3). Cell-based glycan arrays may reach astronomical numbers considering all possible combinations of the glycosylation genes, and organizing distinct sublibraries to cover major steps in glycosylation can help reduce workflows (34, 199). Importantly, cell-based libraries are sustainable once built and may be used not only in direct binding studies, but also with many different applications suitable for cell assays in high throughout (199). The primary use of traditional glycan arrays has been to dissect the binding properties of GBPs (189, 190, 191, 192, 193, 194), and the main advantage of cell-based glycan arrays is the opportunity to dissect interactions in a more natural context using a number of different assays available with cells. One of the first studies employing a small library of human myeloid HL-60 cells designed to dissect contributions of three types of glycoconjugates carrying the SLe^x^ selectin glycan ligands (glycolipids, N-glycoproteins, and GalNAc-type O-glycans by KO of *UGCG*, *MGAT1*, and *COSMC*, respectively) provided evidence that the types of glycoconjugates differentially promoted different phases of the rolling and adhesion cascade of leukocytes (126). A more expanded library of glycoengineered CHO cells was used to explore the glycan-binding specificities of Galectins recapitulating known preferences for N- or O-glycans (200), and studies of two Siglecs employing a library of HEK293 cells revealed distinct binding to different types of glycoconjugates (34).

Importantly, cell-based glycan libraries are providing evidence for more complex interactions with glycans that may involve a higher-order clustering or patterning guided by protein features as originally proposed by Varki (201, 202). The molecular basis for such is still unclear, but, *e.g.*, one illustrative example of a hybrid binding epitope comprised of a glycolipid (GT1b ganglioside) and a cell-membrane protein (synaptotagmin) was recently elucidated for neurotoxin B from *Clostridium botulinum* (203). These complex interactions can be probed by glycoengineering. Coreceptor involvement may also be interrogated. For example, recent findings indicating that the SARS-CoV2 viral spike protein exhibits binding to heparan sulfate (HS) GAGs (204) were explored by KO glycoengineering to demonstrate that HS does serve as a coreceptor strongly impacting binding to ACE2 and infection (205). Cell-based array studies with human and avian influenza virus hemagglutinins (HAs) reproduced the established preferences for α2-6Sia and α2-3Sia (193), respectively. However, these studies also indicated that the human influenza virus HA (Hong Kong/1968 H3) showed preference for N-glycans, while the avian HA showed indiscriminate binding regardless of the type of glycoconjugate (34). We recently developed a cell-based platform for display of the densely O-glycosylated tandem repeat (TR) regions of human mucins using reporter constructs expressed in glycoengineered HEK293 cells, and this array has revealed distinct O-glycan and mucin TR specificities of viral and bacterial adhesins (34).

### 3D organotypic tissue models

**Figure 4:**
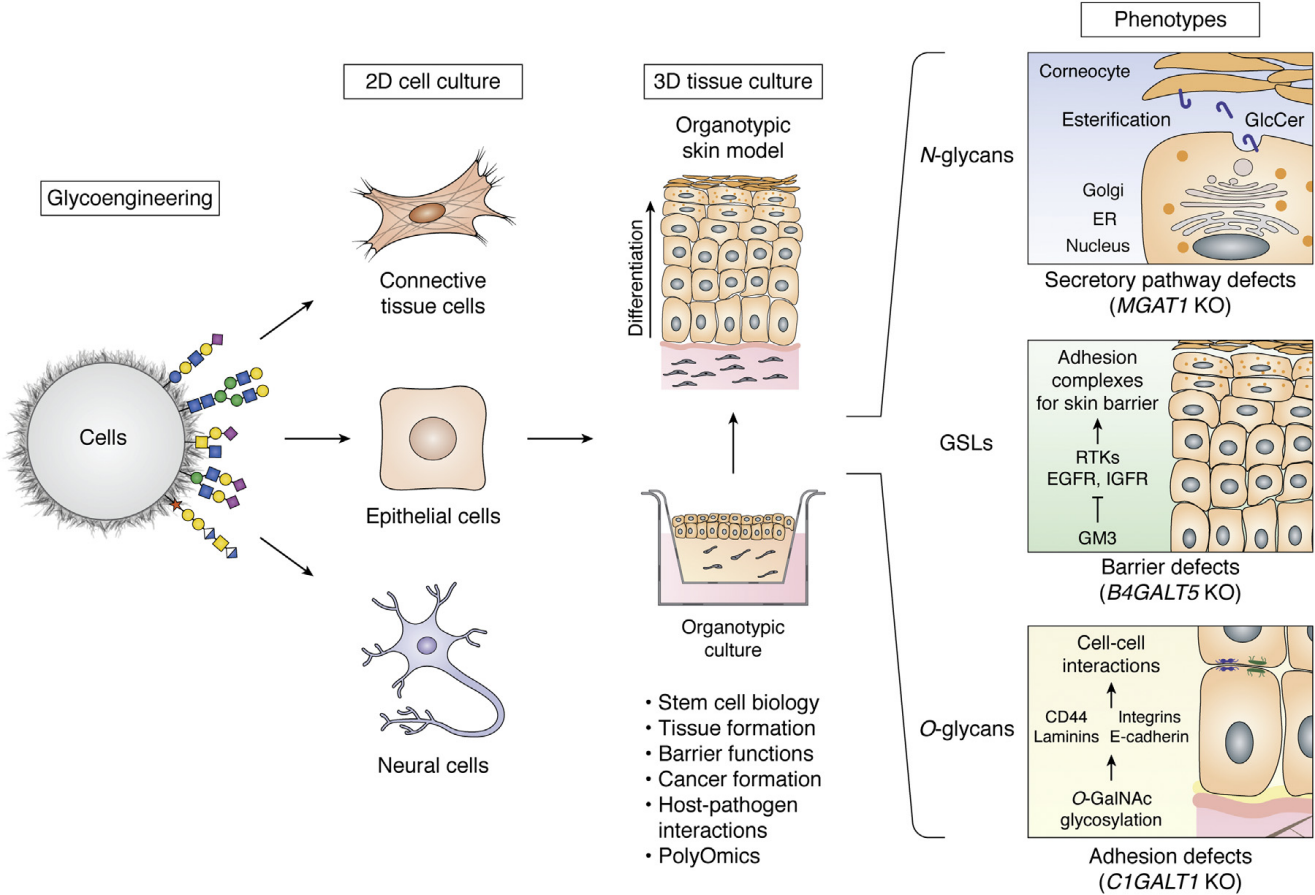
**Dissecting glycan functions using glycoengineered cell lines and organotypic tissue models.** Stable engineering (in 2D) of cell types (*left panels*) such as primary cells, immortalized cell line, induced pluripotent stem cells, or embryonic stem cells, with capability to differentiate into tissue-like 3D structures (*e.g.*, skin models, *right panels*), can be used to explore and dissect biological functions. Genetically engineered organotypic models simplify the interrogation and dissection of biological interactions involving glycans and enable the examination of how the individual glycoconjugates impact stem cells, tissue formation and homeostasis, cellular transformation, the interaction between different cell types, and host–pathogen interaction. For illustration phenotypic characteristics of glycoengineered human organotypic skin models with selective loss of elaborated N-glycans, glycosphingolipids, and GalNAc-type O-glycans are shown schematically (*far right panel*). Loss of complex N-linked glycans (KO *MGAT1*) affects transport and secretion of select proteins, loss of elongated glycosphingolipids (KO *B4GALT5*) affects tyrosine kinase regulation and produces barrier defects, and loss of elongated O-glycans (KO *C1GALT1*) affects differentiation and cell–cell interactions (103). Importantly, global proteomics and genomics can be integrated in the workflow to identify the molecular pathways affected by the loss/gain of individual glycoconjugates (147).

While regulation of the glycome should be studied at the single cell level, the functional cues orchestrated by the glycome clearly have wider roles in tissue organization arguing for use of more complex models. The systematic glycoengineering concept for cell-based glycan arrays can be extended to cell lines and pluripotent stem cells suitable for development of organotypic cultures. Thereby, the glycoengineering strategies employed on 2D cultures can be extended to 3D cultures to explore and dissect biological roles in more complex tissue formation (Fig. 4). This strategy was used with 3D spheres of engineered LS174T colon cancer cells to demonstrate that the repertoire of GALNTs initiating O-glycosylation may contribute to the malignant phenotype. Thus, when the characteristic colon-cancer-associated expression of GALNT6 was replicated in the LS174T model, normal cell differentiation was inhibited (152). Similarly, the characteristic expression of aberrant truncated O-glycans (Tn [GalNAcα1-O-Ser/Thr] and STn [Neu5Acα2-6GalNAcα1-O-Ser/Thr] in cancer was replicated in human keratinocyte cell (HaCaT) human skin models by KO of *COSMC* (private chaperone for the core1 synthase C1GALT1 that controls elongation of O-glycans) and used to demonstrate that the truncation of O-glycans induced oncogenic features including hyperproliferation and invasive growth (206). Another human 3D skin model (*N*/TERT-1) was used to address contributions of elaborated glycans on distinct types of glycoconjugates (glycolipids, N-glycans, O-GalNAc, O-Fuc, O-Glc) by KO targeting of earliest core extension steps (103), as well as probe nonredundant functions of GALNTs isoenzymes (147). Studies in these isogenic cell model systems further provide opportunities to apply wider multiomics approaches to explore changes in the transcriptome and, *e.g.*, phosphoproteome for discovery and dissection of mechanism (147, 206).

### Glycoproteomics

Genetic glycoengineering has also advanced glycoproteomics. A major obstacle in analysis of glycosylation of proteins with proteomics strategies is the need for enrichment of glycopeptides where diversity and heterogeneity of glycans hamper uniform enrichment steps. A cocktail of lectins can enrich most N-glycopeptides (207, 208), and for GalNAc-type O-glycans the Jacalin plant lectin enriches most simple types of O-glycans (core1 based) (209, 210). Other strategies include metabolic labeling with GalNAz or alkynyl fucose for enrichment by click chemistry (211, 212, 213, 214, 215) and selective capture of periodate-oxidized sialic acid containing glycopeptides on hydrazide beads (216, 217). However, genetic glycoengineering can be used to custom design glycosylation to fit to particular enrichment strategies. Simplifying the diversity of glycan structures and eliminating/reducing heterogeneities in structures may be used to enable efficient enrichment. The SimpleCell O-glycoproteomics strategy is based on this concept (127, 218). This strategy was originally employed for GalNAc-type O-glycosylation where KO of either the chaperone *COSMC* or the *C1GALT1* synthase gene was known to abrogate O-glycosylation in most cell lines (except for cells expression the core3 synthase B3GNT6) (219, 220). This leaves all O-glycans truncated to the GalNAcα1-O-Ser/Thr (Tn) structure and/or its α6-sialylated variant (STn) amenable for efficient capture by the VVA and Jacalin lectins (127, 221). The deepest O-glycoproteome analysis to date is with over 3000 O-glycoproteins identified and 15,000 glycosites derived from such SimpleCell lines (26, 119, 127, 221, 222, 223). The SimpleCell strategy was further designed to explore protein O-mannosylation by KO of *POMGNT1* (truncation of core M1 O-glycans to Manα1-O-Ser/Thr) (224, 225), which enabled enrichment by ConA lectin (after prior removal of N-glycans) and discovery of extensive O-mannosylation of the cadherin and plexin superfamilies of receptor proteins (226). Subsequently, KO of the protein O-mannosyltransferases POMT1/T2 resulted in discovery of two new O-mannosylation pathways directed by *TMTC1-4*, and a yet unreported gene dedicated to the cadherin and plexin receptors, respectively (130, 131). Interestingly, KO of *POMGNT1* was in fact not needed for detection of these new O-mannosylated glycoproteins since the initial O-Man residues apparently are not elongated (130) (Fig. 2*A*). Important to note though that the SimpleCell glycoproteomics strategy does not provide information on structures of glycans found at identified glycosites, nor the stoichiometry of O-glycan attachments (221, 227). Recent advances in data-independent acquisition mass spectrometry (MS) may eventually overcome this drawback (222). Genetic truncation of glycosylation pathways in cells could potentially affect the earlier biosynthetic steps because of lack of competition and other unknown factors such as protein interactions (207, 228) and upstream protein functions. However, so far O-glycoproteomics studies performed with SimpleCells and WT cells have not shown substantial differences in identified glycosites (26, 147, 221, 229, 230). Regardless, the use of glycoengineering to custom design glycosylation in order to improve glycoproteomics strategies should be widely applicable and of value in exploring the different glycosylation pathways.

### Cell models for development of enzyme inhibitors

Small-molecule inhibitors of glycosylation enzymes are useful tools to dissect glycan functions in cells and animal models and have great potential for therapeutic applications (20). Global inhibitors of N-glycosylation (231, 232), GalNAc-type O-glycosylation (233, 234), sialylation (235, 236), fucosylation (235), and heparan sulfate biosynthesis (237) have been developed. However, inhibitors often lack selectivity for specific glycosylation enzymes, in particular isoenzymes, or have off-target effects (238). Screening for inhibitors of glycosyltransferases is often performed with *in vitro* assays using recombinant enzymes (233, 239), and it has been a challenge to develop cell-based assays that report direct inhibitors of GTs. Linstedt and colleagues used glycoengineered isogenic cell models to screen for GALNT isoenzyme-specific inhibitors using fluorescence biosensors that selectively report isoform-specific GALNT enzyme activities (furin cleavage of unique peptide substrates) (240). These biosensors were installed in isogenic cells with and without the *GALNT* gene of interest, and the strategy enabled discovery of one compound (T3Inh-1) that selectively binds and inhibits the GALNT3 isoenzyme *in vivo* (241). In another example, the two catalytic subunits of the OST, STT3A and STT3B, were KO engineered to screen and identify selective inhibitors of STT3B enabling regulation of N-glycan sites targeted mainly by posttranslational N-glycosylation (242). These examples demonstrate how glycoengineering of engineered mammalian cell lines now provides cell-based platforms for screening of selective inhibitors of specific biosynthetic steps and isoenzymes.

## Production of therapeutic glycoproteins in glycoengineered mammalian cells

**Figure 5:**
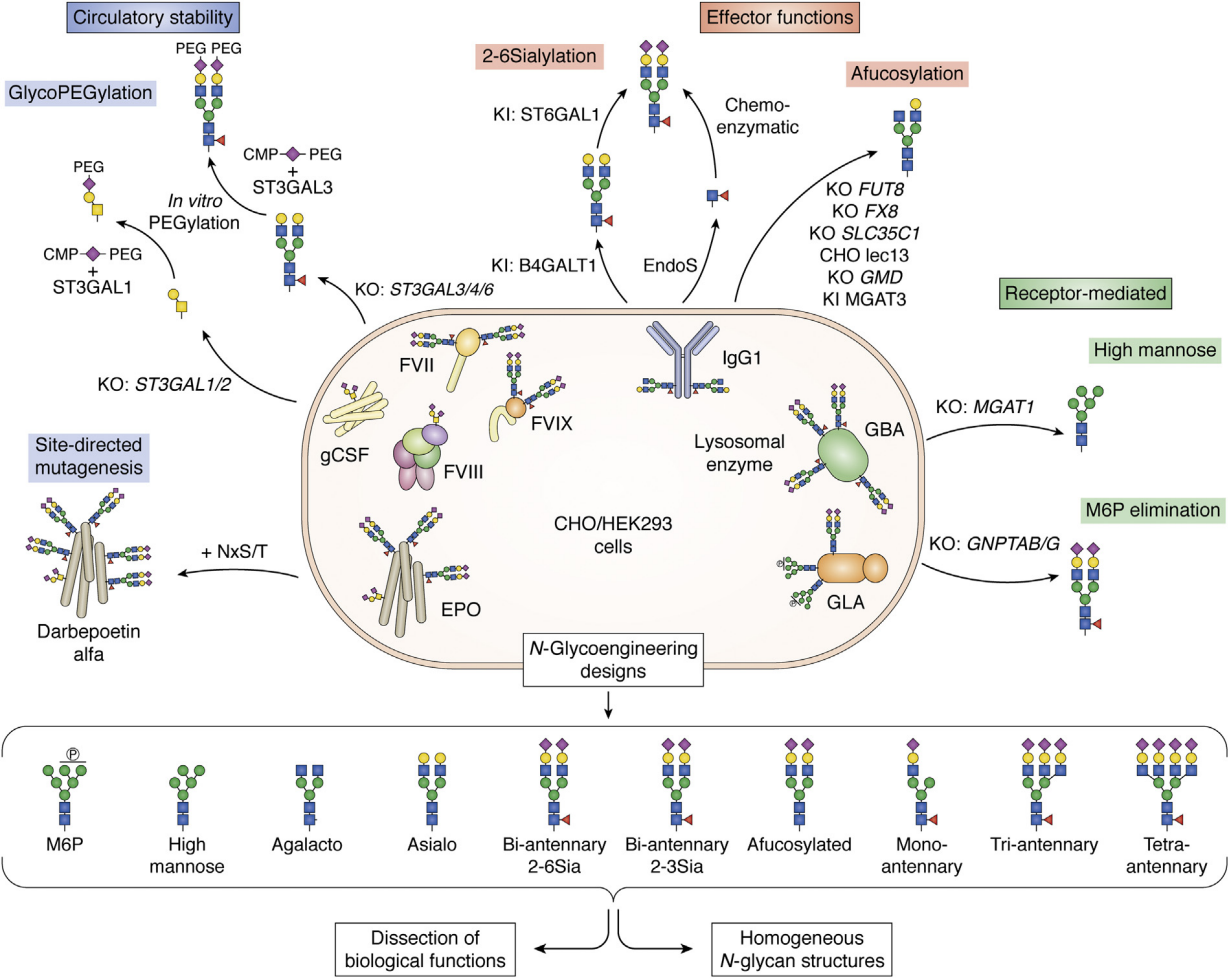
**Glycoengineering strategies for recombinant glycoprotein therapeutics.** Overview of examples of engineering designs applied to N-glycosylation in CHO and HEK293 cells to enhance circulation time of N-glycoproteins, modulate effector functions of therapeutic monoclonal antibodies (IgG1), and redirect lectin-mediated cell uptake of lysosomal replacement enzymes. Combinatorial glycoengineering of CHO and HEK293 cells has enabled production of custom-designed therapeutic glycoproteins with a variety of more homogeneous N-glycan structures that opens up wider screening and exploration of effects on pharmacokinetics, bioactivities, and immunogenicity (*bottom panel*) (82).

The state of current therapeutic biologics seems to constitute a conundrum—most biologics are glycoproteins, yet glycans are not commonly considered in design and development of therapeutics. Glycosylation of therapeutic proteins may influence solubility, stability, structure and hydrodynamic size, clearance, cellular uptake, biodistribution, biological activity, and immunity, yet to day most therapeutic biologics are produced in the classical CHO cell line with its inherent and rather simple, but human-like glycosylation capacities (19, 243, 244, 245). Decades of efforts have been devoted to engineering the glycosylation capacities in yeast (19, 246), insects (247), and plant cells (17, 248), mainly aiming to produce proteins with human-like glycosylation. Glycoengineering in mammalian cells has primarily aimed to reduce the heterogeneity (and improve consistency) in glycosylation (249, 250), increase sialylation to improve circulation (251, 252), and reduce or eliminate the core Fucose residue on IgG antibodies to enhance ADCC (253) (Fig. 5). However, glycoengineering may find much wider use. For example, CHO cells may be engineered to simplify the enzymatic glycopegylation strategy by producing substrate glycoproteins with N- or O-glycans suitable for direct transfer of sialic acids with polyethylene glycol (PEG) chains (254), and proteins may be designed with glycodomain modules and expressed with custom-designed glycans to enhance pharmacokinetics or immunogenicity (255, 256). The success with engineering CHO cells to produce afucosylated IgG therapeutic antibodies clearly stands out as the major achievement and perhaps the only widely adopted glycoengineered mammalian host cell for production of recombinant therapeutics today. Recent reviews cover these advances (16, 20, 257), and here, we limit the discussion to the opportunities provided by glycoengineering using precise gene editing in mammalian cells.

### Therapeutic IgG antibodies

A conserved N-glycan in the Fc region (Asn297) of IgG1 is critical for binding to Fc receptors (258), and the structural features of this N-glycan provide important modulation of interactions with FcγRs and complement factor C1q (259, 260, 261) (Fig. 5). Reducing or removing the core fucose of the Asn297 N-glycan results in increased binding to FcγRIIIa and enhanced ADCC (24, 262, 263, 264). This insight has been used to produce therapeutic antibodies with improved cytotoxicity in CHO cells overexpressing MGAT3 to inhibit core fucosylation (265), and in CHO cells with KO of the *fut8* gene that nonredundantly regulates core fucosylation (24, 25, 253, 266, 267, 268, 269). The first CHO cell line engineered with *fut8* KO was generated by targeted homologous recombination (24), which is now readily reproduced with precise gene-editing strategies (270). Moreover, KO of other enzymes and transporters in *de novo* fucose biosynthesis (*GMD*, *FX*, *SLC35C1*) have enabled production of afucosylated mAbs in CHO cells (271, 272, 273). Another approach has been to express the prokaryotic GDP-6-deoxy-D-lyxo-4-hexulose reductase (RMD) to interfere with GDP-Fuc synthesis in CHO cells, which has resulted in a tunable system (GlymaxX Technology, ProBioGen) to produce IgG with different levels of α6Fuc (274).The Asn297 N-glycan is sterically hindered by the protein backbone, and as a result the N-glycan is incompletely processed with the predominant structures being biantennary (G0F, G1F, G2F) with low galactosylation and sialylation (275). However, the fine structure of the Asn297 N-glycans appears to be dynamically regulated and correlates with diseases including inflammation, autoimmunity, infections, cancer, and following vaccination (261, 276, 277, 278, 279). The N-glycan structure also affects the pharmacokinetic properties (280). Terminal galactose is reported to increase FcγRIIIa and C1q interactions resulting in enhanced ADCC and complement-dependent cytotoxicity (CDC) (281, 282). Sialic acid capping, in particular α2-6Sia, is indicated to decrease binding affinity of mAbs to FcγRIIIa (283) as well as C1q (284). This may mediate anti-inflammatory effects (285) and improve serum half-life (252), although contradicting studies report no effects of sialic acid on Fc conformation and recognition (286, 287). Despite this there appears to be fruitful grounds for further engineering of Fc glycosylation on therapeutic IgGs; however, given the complexity of the fine-tuning of IgG functions by N-glycosylation, this may not be straightforward and hence difficult to solve by generating simple homogeneous glycoforms. To this end another elegant strategy for fine-tuned control of IgG N-glycosylation through installation of inducible expression of glycosyltransferase genes (*FUT8*, *B4GALT1*) has been developed (288). The engineering options for the IgG Asn297 N-glycan by simple KO/KI combinations of most of the GTs involved in N-glycan branching, galactosylation, and sialylation in CHO cells have been systematically explored (82, 289) (Fig. 5). This provides engineering designs for IgG with homogeneous biantennary N-glycans with and without galactose and fucose, although efficient sialylation (α2-3 and α2-6Sia) is only achievable on the α3-antenna. However, a monoantennary glycoform with homogeneous sialylation was achievable, and further studies are needed to evaluate effector functions of this (289). A different strategy—GlycoDelete—to simplify the IgG1 N-glycan to simple sialylated trisaccharides was engineered in HEK293 cells by KO of *MGAT1* to produce high-Man glycans combined with installation of a bacterial endo-β-N-acetylglucosaminidase (EndoT) to truncate high-Man N-glycans (290). IgG with these short N-glycans exhibits prolonged circulation time and reduced FcR binding, demonstrating the potential of glycoengineering for production of therapeutic IgG with homogeneous and custom-designed glycosylation.

### Custom-designed glycosylation of recombinant therapeutic glycoproteins

While IgG represents a unique challenge for glycoengineering due to the protein-specific restricted glycosylation, erythropoietin (EPO) is a widely studied therapeutic glycoprotein that acquires glycosylation according to the host cell glycosylation capacity (82, 291). The three N-glycans on EPO acquire rather homogeneous tetra-antennary structures with a minor amount of polyLacNAc and efficient α2-3Sia capping when produced in CHO cells (292, 293), while a single sialylated core1 O-glycan is heterogeneously attached (∼80% occupancy) (294). EPO stimulates red blood cell production and is widely used in treatment of anemia, but the native glycoprotein is rapidly cleared by glomerular filtration due to its small size (30 kDa). Pioneering work led to new generations of EPO with extended circulation, with one strategy involving introduction of two additional N-glycans (Darbepoetin alfa) to increase the hydrodynamic size and prevent clearance (295). The clearance of EPO is also highly sensitive to the sialylation state (292, 296), and sialic acid capping of glycans on glycoproteins is generally important for the serum half-life to prevent lectin-mediated uptake by the hepatic Ashwell–Morell receptor (AMR) (297, 298, 299, 300). Batch-to-batch variations in sialylation of EPO have been a challenge (301), and secreted neuraminidases (*NEU1-3*) from CHO cells can pose issues and KO of these can improve sialylation (302). Several reports have suggested that CHO cells may add minor amounts of the immunogenic α1-3Gal epitope as well as NeuGc sialic acids (303, 304, 305), although these findings are disputed and CHO cells with KO of these genes are available (306).

The N-glycosylation capacity of CHO cells was extensively deconstructed by KO and to some extend reconstructed by limited KI of human GT genes (82, 121). Using EPO as the N-glycoprotein reporter, these studies demonstrated wide design options for antennary structures, galactosylation, polyLacNAc and α2-3/2-6 sialylation, and the ability to produce more homogeneous glycoforms (82) (Fig. 5). Redundant contributions of the many B4GALT and ST3GAL isoenzymes for efficient galactosylation and α2-3 sialylation, respectively, necessitate combinatorial KO to eliminate either of these processes (82, 185). N-glycoengineering may not be generally applicable to all N-glycoproteins and especially individual N-glycosites, since local conformational effects may influence glycosylation at some glycosites, as discussed for IgG above. Individual N-glycosites may undergo selective processing, *e.g.*, high-Man N-glycans are often found at individual glycosites in proteins, and those have to be considered and specifically addressed in glycoengineering. Regardless, the current state of glycoengineering in mammalian cells provides considerable freedom to design and control most types of glycosylation of proteins, which provides the opportunities to produce glycoprotein variants and test these to identify the optimal design.

### Glycodesign options for enzyme replacement therapies

Lysosomal enzymes are N-glycoproteins and select N-glycans on these acquire mannose-6-phosphate (M6P) in early Golgi, which serves as recognition for the mannose 6-phosphate (M6P) receptors (MPRs) mediating trafficking from Golgi to the lysosome (307, 308). Lysosomal storage diseases (LSDs) including Gaucher disease with deficiency in β-glucocerebrosidase (GBA) and Fabry disease with deficiency in α-galactosidase A (GLA) are currently treated by enzyme replacement therapies (ERTs) (308, 309, 310, 311, 312). The MPRs (IGF2R) also serve in endocytosis from the cell surface, and this is exploited in ERTs for uptake and delivery (309, 311, 313). When lysosomal enzymes are expressed recombinantly in mammalian cells, select N-glycans acquire M6P tags, while other N-glycans may become complex-type sialylated glycans, and the degree of M6P tagging is considered the major contribution factor for cellular uptake (309, 311, 314, 315). Several lectin receptors including the mannose receptor (MR) and AMR may also affect uptake and delivery (311, 316, 317), and the first ERT for Gaucher disease utilized this to selectively target macrophages expressing the MR by trimming N-glycans including M6P to the mannose core with enzyme digestion (318). This glycan design has also been engineered directly in CHO cells (121). However, engineering of glycans on ERTs has mainly focused on increasing M6P for improved delivery (311, 319), and systematic studies of the optimal glycan features were only performed recently using extensive combinatorial glycoengineering in CHO cells (121) (Fig. 5). Testing a panel of glycoforms of GLA *in vivo* in a Fabry mouse model revealed that M6P is not necessary for uptake and reduction of substrates in target organs, and interestingly GLA with α2-3Sia N-glycans exhibits higher circulation time than GLA with M6P (*e.g.*, Fabrazyme), while GLA with α2-6Sia bi-antennary N-glycans is rapidly targeted to the liver. This example illustrates that testing different glycoforms of glycoproteins may be a fruitful strategy to improve and optimize design of therapeutic glycoproteins.

### Native mass spectrometry of glycoproteins

Heterogeneous glycans pose challenges for analysis of intact glycoproteins by MS (320, 321), and considerable efforts are devoted to characterization of therapeutic glycoproteins by bottom-up MS analysis of peptide digests and profiling of released glycans. Native MS is widely used to study noncovalent protein–protein complexes, and direct MS of intact proteins is also highly suitable for analysis of the quality of recombinant proteins with posttranslational modifications (322). Native MS was applied to CHO cell produced IgG that carries the rather simple N-glycan structure with limited heterogeneity at Asn297 (323, 324). MS analysis of more complex glycoproteins with multiple N- and O-linked glycans has relied on exoglycosidases to reduce glycan heterogeneity to enable data interpretation (325, 326). However, glycoengineering of production host cells offers simpler ways to obtain glycoproteins with more homogeneous glycans suitable for intact MS analysis (82), and this strategy was used with multiple glycoforms of EPO produced in glycoengineered CHO cells (327). We envision that glycoengineering will greatly advance use of MS analysis of intact glycoproteins not only in basic science, but also in quality control of glycoprotein therapeutics.

## Conclusion and prospective

In 2014, we argued that the emergence of precision gene-editing technologies represented a small revolution for the glycobiology field—if anything an understatement (4). Precision engineering of cellular glycosylation is becoming indispensable for interrogation and dissection of biological interactions involving glycans. Arguably, today it may be simpler and more informative to gain information on the genes and biosynthetic pathways that regulate glycosylation, than to directly analyze structures of glycans. Tools and expertise for genetic engineering of cellular glycosylation are widely available to cell biologists, and the workflows employed may be integrated with general proteomics and genomics approaches. There are clearly still shortcomings that need to be addressed, and the most critical ones are to improve knowledge of the *in vivo* functions of the many GT isoenzymes and develop strategies for translating quantitative transcriptomics and proteomics data into reliable predictions of the cellular glycome. Genetic glycoengineering is currently the only method to study glycosylation at the single cell level.

Genetic glycoengineering is bringing new tools and capabilities to the glycomics field that provide innovative platforms for exploring functions of glycosylation in the natural environment of the cell such as cell-based glycan arrays and organotypic tissue models. These platforms are still being developed and knowledge of their use is limited, but results obtained with these are already providing insights into more complex interactions of GBPs with higher-order patterns of glycans on proteins. Revisiting the binding properties of glycan-binding proteins with these new resources is likely to be fruitful and adds insights into how selectivity of interactions with glycans is obtained.

Glycoengineering is poised to transform the design and production platforms for recombinant glycoprotein therapeutics. While implementation of glycoengineered host cells in the conservative pharma environment will take time, the advantages of being able to produce more homogeneous glycoforms with the added value of enabling intact analysis of biomolecules are too good to miss. We envision that the major incentive for glycoengineering of therapeutics will come from discovery of glycan designs that provide improved properties to glycoprotein therapeutics such as exemplified by afucosylated IgG therapeutic antibodies. This will require experimental testing, and it is now possible to produce and display a variety of different glycoforms of glycoproteins for use in preclinical studies.

Finally, beyond engineering of glycosylation, the precise gene-editing tools open up an entirely new level for exploring the glycosylation machinery in live cells. For example, tagging of endogenous GTs can enable live imaging of subcellular localization and changes herein in response to stimuli as well as molecular interactions. Tagging endogenous protein substrates can also be used to monitor protein-specific glycosylation in real time revealing effects on subcellular transport. In conclusion, genetic engineering of glycosylation is a fertile playground with enormous potential for reprogramming the glycosylation processes in cells and the cellular glycome for research and therapeutic applications.

## Conflict of interest

University of Copenhagen has filed a patent application on the cell-based display platform. GlycoDisplay Aps, Copenhagen, Denmark, has obtained a license to the field of the patent application. Y. N., Z. Y., and H. C. are cofounders of GlycoDisplay Aps and hold ownerships in the company. H. C. is a cofounder of GO-Therapeutics Inc and holds ownership. H. W. is a consultant for GO-Therapeutics Inc and cofounder of EbuMab ApS and holds ownership.
